# The German National Action League for people with rare diseases: translating the three tiers center system into active co-operation, a one center experience

**DOI:** 10.1186/s13023-019-1130-5

**Published:** 2019-06-27

**Authors:** U. Plöckinger, A. Ziagaki

**Affiliations:** Center of Excellence for Rare Metabolic Diseases, Interdisciplinary Center of Metabolism: Endocrinology, Diabetes and Metabolism, University-Medicine Berlin, Campus Virchow-Klinikum, Augustenburger Platz 1, 13352 Berlin, Germany

**Keywords:** Rare diseases, National action plan, Co-operation

## Abstract

**Introduction:**

In 2009 the European Commission called for National action plans (NAP) to improve the care for persons with rare diseases. Germany set up a NAP in 2013 suggesting a three-tiered structure of co-operating centers (CC), centers of excellence (CE) and reference centers (CR). Since then CEs and CRs were organized in the framework of university hospitals. However, realization of CCs taking into account the requirements of the NAP has been slow. We therefore set-up a 12-months program to initiate co-operation and to support the development of structured CCs.

**Methods:**

Our center invited 3000 physicians from Berlin and/or Brandenburg to participate. They were chosen either due to already referring patients with rare metabolic diseases to the center, residing in a neighborhood with diverse ethnic background, known to have a high prevalence for specific metabolic diseases, or working as a medical sub-specialist (gastroenterology, hematology, rheumatology) with a high probability to diagnose a rare metabolic disease. The center offered co-operation contracts, administrative and structured medical support, privileged access to the center for physicians and their patients, as well as a program of continuous medical education (CME) over a period of 12 months.

**Results:**

Between 0.1 to 0.5% (mean 0.2%) of the invited physicians participated in CME meetings. None of them was interested in setting up a co-operating center. The physicians were interested in broadening their knowledge about rare diseases, but less so in direct care for these patients and not at all in fulfilling the requirements of the NAP.

**Conclusions:**

The requirements of the NAP for CC are thought of as unrealistic due to their demands on structural re-organization, quality measurements and additional work-load for outpatient-care. Especially so, with respect for the low number of patients profiting from these efforts and the lack of re-imbursement. We suggest a reconsideration of the German NAP.

## Background

In 2009 the European Commission published a council recommendation to set up national plans to improve the medical support of patients with rare diseases [1]. The European Council on an Action in the Field of Rare Diseases recommends that Member States “identify or create appropriate centers of expertise, foster the participation of national centers of expertise in existing European reference networks, create structures that facilitate the cooperation of specialists and the exchange of experts and expert knowledge in this area, on both the national and international level, create the requirements for the dissemination of the expert knowledge necessary to treat rare diseases patients locally and encourage the treatment of all persons with rare diseases to be based on a multidisciplinary approach.” Subsequently the German Federal Ministry of Health (BMG) together with the German Federal Ministry of Education and Research (BMBF) and the Alliance for Chronic Rare Diseases (ACHSE e.V.) founded the National Action League for People with Rare Diseases (NAMSE). The goals were to enable a concerted effort, prepare suggestions for a National Action Plan for People with Rare Diseases and support the establishment of national centers of expertise [2]. In 2013 the National Plan of Action for People with Rare Diseases was published by the National Action League for People with Rare Diseases (NAMSE). The report describes the overall health situation of persons with rare diseases, the action fields and goals of a National Plan of Action. Paragraph 3 details the center model for rare diseases [2]. NAMSE recommends the establishment of centers for rare diseases at three different, cross-linked levels of specialization. Type C centers [co-operating centers (CC) i.e. non-hospital subspecialized practices, group practices, medical care centers or hospitals] are to be responsible for one specific disease or disease-group offering interdisciplinary, multi-professional outpatient care. They deliver care for patients with a confirmed diagnosis or a clear suspected diagnosis. Type B centers [centers of expertise (CE) for a specific rare disease or disease group] offer outpatient and inpatient, interdisciplinary and multi-professional care. Type A centers consist of more than two type B centers and offer special non-disease specific structures (e.g. for the treatment of patients with unclear diagnoses, patient guides, interdisciplinary case conferences, innovative special diagnostics). These type A centers [reference centers (RC)] are the referral center for patients with an unclear diagnosis; they do basic and clinical research, provide training and -tools covering the medical dimension of care for undergraduate medical students. The National Plan of Action suggests to set up a central body for the certification of centers [2]. For a detailed list of criteria to be met in the three-tiered center model see Appendix 4.2 of the NAMSE action plan [3]. In 2018 the certification process has not yet been installed. Responding to the NAMSE proposals “centers of expertise” have been set up since 2013 mostly at university hospitals.

**Table 1:** Participants

Presentations	Attendees/Enrolled	Medical sub-specialities (*N*)
Kick-off meeting and presentation on rare metabolic diseases	15/30	Internal medicine (10), general practitioner (2), neurology (3)
M. Gaucher	3/10	Internal medicine (3)
Pompe Disease	3/ 8	Internal medicine (1), neurology (2)
Mucopolysaccharidosis	2/ 5	Internal medicine (2) Internal medicine (3), paediatric
Phenylketonuria	14/25	medicine (2), neurology (1), dietitian (8)^a^

^a^ None of the dietitians had been officially invited, and thus were not calculated in the percentage of physicians responding

**Fig. 1:**
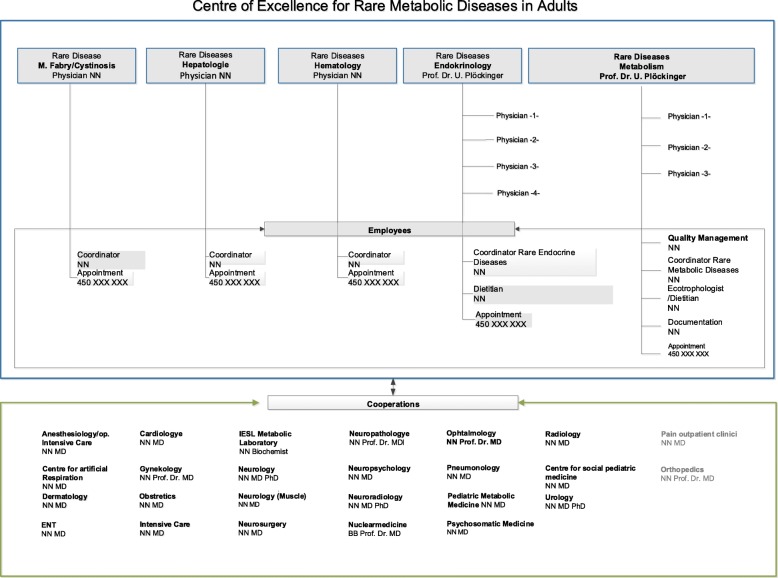
Organigram Center of Excellence Rare Metabolic diseases in Adults

The Center of Excellence for Rare Metabolic Diseases in Adults at the Charité university-medicine in Berlin is one of those centers matching the NAMSE requirements for type B Centers (Fig. 1). In addition, the center takes on many of the responsibilities of a type A Center, including treatment of patients with unclear diagnosis, patient guides, case conferences, basic and clinical research, continuous medical education for rare metabolic diseases for physicians and medical students. While standards for the certification for the NAMSE centers are still lacking the center built its own quality-management infra-structure and was certified according to ISO DIN 9001–2008/2015. In addition, the Center of Excellence for Rare Metabolic Diseases in Adults is part of the European Reference Network MetabERN.

In 2016 the Center of Excellence for Rare Metabolic Diseases in Adults initiated a project to cooperate with non-hospital subspecialized practices for the care of patients with inherited metabolic diseases. The main goals of this cooperation were defined as

- establishing a co-operation between general practitioners, subspecialised outpatient practices according to the criteria set up by NAMSE, in an effort to set up CC.
- offering patients standardized care close to their home [1]
- increasing the awareness for rare metabolic diseases with the participating physicians.

The program was scheduled with 5 meetings in the course of 12 months. Here we report on the details of the project and the results achieved.

## Methods

The co-operation was planned as suggested in the NAMSE action plan. Specifically, the co-operating physicians (CC) were supposed to care for a patient with a rare metabolic disease in close cooperation with the CE. To facilitate this co-operation the CE offered to provide

- all standard operating procedures i.e. clinical pathway recommendations on diagnosis, treatment and follow up for the relevant diagnosis, emergency or specific care (i.e. pregnancy)
- matrices for standardized documentation

### References and literature

- access to its electronic patient files (KIS) after the respective patient agreement and
- responsibility of the necessary data protection.

To further facilitate the cooperation the CE offered

- a telephone hotline with direct access to the CE’s physician, as well as
- privileged access of the CC’s patients to the CE and thus reducing the waiting time for appointments.

To facilitate the implementation of the NAMSE criteria the physicians were offered

- continuous medical education (CME) 5 times within the 12 months period.
- members of the CC were invited to participate in the CE’s internal CME set up for its own medical personal (nurses, dietitian, administrative personal) every 4 weeks.

In addition to the co-operation based on the suggestions by NAMSE the CC was offered a contract on co-operation with the Charité University Hospital. The contract of co-operation referred to the CC as “Partner of the Charité”, and public use of this label was possible.

### Participants

The CE invited 3000 physicians in Berlin to participate. Of these 623 (21%) had already referred patients with an inherited metabolic disease and 2250 (75%) of the invitees had co-operated within the last five years with the Interdisciplinary Center of Metabolism: Endocrinology, Diabetes and Metabolism, i.e. the department that includes the CE for Rare Metabolic Diseases in adults. Twenty five percent of the invited physicians were situated in a part of Berlin with specific ethnic populations (Turkish, Arabic and Jewish) with a high prevalence of rare diseases, or were subspecialised in disciplines that were supposed to have a high probability to diagnose symptoms of rare metabolic diseases (gastroenterology and haematology with respect to splenomegaly and M. Gaucher, neurology with respect to muscle symptoms and Pompe disease, rheumatology with respect to dysostosis multiplex and mucopolysaccharidosis).

### Program

All 3000 physicians were invited to participate in a kick-off meeting. There the National Action Plan for People with Rare Diseases was presented, followed by a presentation of the details of the suggested cooperation. All attendees received printed copies of the presentation, the suggested contracts, the workflow of the cooperation, as well as the time-table for further meetings. All were informed how to get in contact with the Center in case of need for further information or consent on the suggested co-operation and/or signing the contract.

Thereafter four additional CME meetings were scheduled discussing one specific disease each: M. Gaucher, Pompe disease, mucopolysaccharidosis and phenylketonuria.

After each presentation the CE discussed with the attendees their interests in rare diseases, their ideas on co-operation and possible rationales against participating in the suggested co-operation. The results of these discussions were documented and were thus available for the overall evaluation of the program.

All five meetings offered a multiple-choice questionnaire with at least 10 questions on the topics of the presentations, which if filled-out would gain an additional CME point. All meetings were evaluated using a standardized questionnaire from the Berlin Chamber of Physicians.

## Results

Fifty percent of those enrolled (15/30) for the kick-off meeting attended, thus 0.5% of invitees responded to the invitation on co-operation. For details on the following meetings and the medical sub-specialisation of the attendees see Table 1. None of the physicians who had already referred patients with rare metabolic disease to the CE participated. Although all participants stated they were interested on information none was willing to enroll in the co-operation program. The reasons were in the order of frequency: they did not care for patients with rare metabolic diseases (*N* = 15), the administrative effort to set up a contract on co-operation with the Charité was not considered worthwhile in respect to possible gains (N = 15), the time needed to set up the administrative organisation for the qualification as a NAMSE co-operation center was considered time lost (*N* = 12). All attendees stated they were interested in the medical CME program as it offered improvement of their knowledge on rare diseases, they wanted to get in personal contact with the center’s physician and were eager to know the CE’s organisation. Access to the center via a telephone hotline was not considered necessary, as all already had had contact with the center and described the access to the center’s physician as uncomplicated. Similar reasons were given for declining a privileged access for their patients to the CE, referring to earlier experience with the center as co-operative and fast responding in case of emergency.

After a twelve months period of offering co-operation and support for setting up a CC according to the NAMSE criteria to general practitioners and/or subspecialised outpatient practices not one of those interested took advantage of the offer.

## Discussion

In 2013 the National Plan of Action for People with Rare Diseases had been published, suggesting the organization of a three-tiered center structure for the care of persons with rare diseases. Since then CE and CR have been set-up. Most of them were related to or were part of a university hospital. In contrast the development of CCs has been difficult. The CE for Rare Metabolic Diseases in Adults at the Charité, University-Medicine Berlin undertook to support the organization of CCs with a group of 3000 physicians, selected according to their probability of diagnosing a patient with a rare metabolic disease or known to refer such a patient to the CE. The co-operation program offered most of the necessary administrative and organisational basics asked for by the National action plan., The center provided the respective standard operating protocols, documentation matrices, literature and a CME program. To increase attractivity of the program a contract with the Charité was offered, as well as privileged telephone contact (hotline) and patient access. Between 0.1 to 0.5%% (mean 0.2%) of those invited participated in the CME meetings. However, none of those attending was convinced to profit from the suggested cooperation. The arguments against such a standardized co-operation are easily understood. Despite the offer of the CE to provide most of the medical and administrative material and thus the basic structure for the co-operation, the administrative efforts needed to get certification by NAMSE are time- and resource consuming. Especially so if related to the number of patients concerned. At the time of the meetings none of the participants cared for a patient with rare disease. The catalogue of quality measure to be undertaken for a CC to be certified by NAMSE in the near future was not aligned with the quality management used by the participants. The extend of the documentation and the participation in research were considered unrealistic. Furthermore, up to 2018 no financial compensation has been agreed upon with the health insurance companies for the time-consuming care of patients with rare diseases.

The experience on informal co-operation with the Interdisciplinary Center of Metabolism: Endocrinology, Diabetes and Metabolism, of which the CE is part of, was considered satisfactory. Telephone contact with the physicians of the Center, communication, patient access in emergencies was judged to be excellent and thus there was no need for an additional privileged access. None of the physicians who already cooperated with the CE on rare disease patient(s) participated in the meetings. Apparently, they saw no necessity to improve the procedures.

**In conclusion**, the development of CC cooperating with CE in the care of these patients is unrealistic. Due to the perceived high-level requirements in the structure and quality management of the CC and the lack of financial re-imbursement any such co-operation is unlikely in the near future. While interest in and awareness of rare diseases are increasing most physicians caring for such patients on an outpatient basis are happy to refer these patients to the CE as their only and primary part in the care for patients with rare metabolic diseases.

## Data Availability

The datasets used and/or analyzed during the current study are available from the corresponding author on reasonable request.

## Abbreviations

ACHSE: Alliance for Chronic Rare Diseases
BMBF: German Federal Ministry of Education and Research
BMG: German Federal Ministry of Health
CC: Co-operating Centers
CE: Centers of Excellence
CME: Continuous Medical Education
CR: Reference Centres
KIS: Krankenhaus Informationssystem (Hospital Patient Software)
NAMSE: National Action League for People with Rare Diseases/National Plan of Action for People with Rare Diseases
NAP: National Action plan
